# Parents make the difference: a randomized-controlled trial of a parenting intervention in Liberia

**DOI:** 10.1017/gmh.2015.12

**Published:** 2015-08-04

**Authors:** E. S. Puffer, E. P. Green, R. M. Chase, A. L. Sim, J. Zayzay, E. Friis, E. Garcia-Rolland, L. Boone

**Affiliations:** 1 Department of Psychology and Neuroscience, Duke University, Box 90086, 417 Chapel Drive, Durham, NC, USA; 2 Duke Global Health Institute, Box 90519, Durham, NC, USA; 3 Center for Child & Family Health, Duke University Medical Center, 1121 W. Chapel Hill Street, Suite 100, Durham, NC, USA; 4 International Rescue Committee, 122 East 42nd Street, New York, NY, USA; 5 International Rescue Committee, Monrovia, Liberia

**Keywords:** Abuse prevention, Africa, family-based intervention, global mental health, Liberia, parenting

## Abstract

**Background.:**

The objective of this study was to evaluate the impact of a brief parenting intervention, ‘Parents Make the Difference‘(PMD), on parenting behaviors, quality of parent-child interactions, children's cognitive, emotional, and behavioral wellbeing, and malaria prevention behaviors in rural, post-conflict Liberia.

**Methods.:**

A sample of 270 caregivers of children ages 3–7 were randomized into an immediate treatment group that received a 10-session parent training intervention or a wait-list control condition (1:1 allocation). Interviewers administered baseline and 1-month post-intervention surveys and conducted child-caregiver observations. Intent-to-treat estimates of the average treatment effects were calculated using ordinary least squares regression. This study was pre-registered at ClinicalTrials.gov (NCT01829815).

**Results.:**

The program led to a 55.5% reduction in caregiver-reported use of harsh punishment practices (*p* < 0.001). The program also increased the use of positive behavior management strategies and improved caregiver–child interactions. The average caregiver in the treatment group reported a 4.4% increase in positive interactions (*p* < 0.05), while the average child of a caregiver assigned to the treatment group reported a 17.5% increase (*p* < 0.01). The program did not have a measurable impact on child wellbeing, cognitive skills, or household adoption of malaria prevention behaviors.

**Conclusions.:**

PMD is a promising approach for preventing child abuse and promoting positive parent-child relationships in low-resource settings.

## Introduction

Children and adolescents worldwide experience high rates of verbal, physical, and sexual abuse, often perpetrated by their caregivers. Young people in certain parts of the world seem to be at increased risk, with multi-country studies documenting the highest rates in Africa (Akmatov, 2011; Stoltenborgh *et al.* 2011). Rates of family conflict, abuse, and a range of poor developmental outcomes seem to be even higher in post-conflict settings where children and families are faced with exposure to community-based violence and other risk factors for family stress and mental health problems, including poverty, displacement, loss of or separation from loved ones, and uncertainty about the future (Mels *et al*. 2010; Reed *et al*. 2012). Daily stressors experienced in post-conflict settings may have as much, or potentially more, effects on mental health as war-related stress. Some findings suggest that family violence is a major predictor of mental health outcomes (Miller & Rasmussen, 2010; Panter-Brick *et al*. 2011), perhaps even more predictive of children's mental health problems than exposure to war violence among children (Miller & Rasmussen, 2010). Thus targeting a broader range of stressors, including mental health and family well-being, may buffer the harmful effects of conflict (Bolton & Betancourt, 2004; Akinsulure-Smith & Smith, 2012).

Child abuse has been associated with lasting emotional, behavioral, physical, and cognitive problems (Moylan *et al.* 2010; Mills *et al*. 2011; Widom *et al*. 2012). Overly harsh and inconsistent parenting, with or without abusive behavior, are also associated with lower self-esteem and social skills, as well as higher rates of anxiety and disruptive behavior (Boudreault-Bouchard *et al*. 2013; Yap *et al*. 2013; Uji *et al*. 2014). In post-conflict settings, harsh parenting may even moderate the effects of exposure to war on children's mental health (Catani *et al*. 2008; Miller & Rasmussen, 2010). Parents who mistreat their children typically demonstrate limited positive interactions with their child, poor understanding of child development, low tolerance of misbehavior, and ineffective discipline strategies. Behavioral parenting programs address these risk factors by increasing skills for positive parent–child interactions and effective discipline and by providing parents with education on child development and behavior (Kaminski *et al*. 2008).

Parenting interventions that address abuse and evidence-based child and family psychosocial interventions in general, are scarce or non-existent in many low-income countries and particularly in conflict-affected settings (Kakuma *et al*. 2011; Kieling *et al*. 2011). The most commonly available services provide general psychosocial support, often using approaches that have not been empirically validated or grounded in evidence-based practices (Tol *et al*. 2011). Prevention and early interventions are needed at the family and community levels to reduce abuse and foster positive parenting practices – protective factors that can buffer against mental health problems and other negative developmental outcomes (Engle *et al*. 2007; Betancourt & Khan, 2008; Kieling *et al*. 2011).

### Behavioral parenting interventions

Empirical evidence suggests that behavioral parent training programs can improve a range of positive caregiver and child outcomes, including child internalizing and externalizing symptoms, parent-child attachment, parenting stress, and parental self-efficacy (Eisenstadt *et al*. 1993; Chase & Eyberg, 2008; Eyberg *et al*. 2008). These programs are grounded in behavioral theory; parents are taught to use positive reinforcement strategies, such as praise, to increase appropriate child behaviors and consistent, non-corporal discipline strategies. The goal is to provide parents with concrete techniques to decrease harsh parenting, improve interactions, encourage child prosocial behavior, and promote positive child development. Among interventions backed by the strongest evidence for use with young children are Triple P (Nowak & Heinrichs, 2008), Parent–Child Interaction Therapy (Eisenstadt *et al*. 1993), Incredible Years (Webster-Stratton *et al*. 2011), and the Nurturing Parenting Program (Maher *et al*. 2011). The magnitude of effects across these varies based on the target population and version of the program. While all primarily behavioral, these programs have unique elements in content and delivery. For delivery, they each require different combinations of inputs, such as varying training models, facilitator requirements, or materials; this influences the settings for which they are appropriate and the degree to which adaptations to implementation plans are required across contexts.

Most of the research supporting parent training programs has been in high-income countries. Of the limited studies in low-income countries, few have targeted reductions in harsh parenting or prevention of emotional and behavioral problems (Klein & Rye, 2004; Jin *et al*. 2007; Oveisi *et al.* 2010; Mejia *et al*. 2012), while more have focused on caregivers of infants and children under age 3 and outcomes related to early mother–child interactions (Knerr *et al*. 2013). Further research is needed to establish the feasibility and efficacy of such interventions in low income, conflict-affected settings and to determine the best strategies for implementation and wide-scale dissemination.

### Current study

We add to the growing evidence based on parenting interventions designed for low-resource settings by conducting a randomized trial of a 10-session program for caregivers of young children in post-conflict Liberia called ‘Parents Make the Difference’ (PMD). PMD was developed for caregivers of children aged 3–7 years. The program is grounded in behavioral theory and shares core concepts with parenting programs backed by the strongest evidence from high-income countries. The content was designed to be culturally relevant and appropriate for lay providers to deliver. We hypothesized that the program would be feasible with limited resources, reduce harsh discipline, increase positive parenting, and improve caregiver–child interactions. Secondary outcomes related to child cognition, emotional and behavioral well-being, and caregiver-child communication. Another program objective was to promote malaria prevention behaviors, particularly caregivers using bednets for their children, as malaria continues to be a leading cause of child morbidity and mortality (National Malaria Control Program *et al*. 2012).

## Methods

### Setting and participants

This study was conducted in Lofa County, Liberia. During the Second Liberian Civil War (1999–2003) that killed between 150,000 and 200,000 people, many residents of this county fled to neighboring Guinea and Sierra Leone. The International Rescue Committee (IRC), an international humanitarian organization, implemented the PMD program approximately 10 years after refugees returned home to rural Lofa County.

The IRC recruited caregivers from five communities in Lofa. Program staff conducted information sessions at schools and invited families to register. To be eligible, adults had to be a caregiver to a child aged 3–7 years entering the first year of formal schooling. If a caregiver had more than one eligible child, staff enumerated all eligible children in the household and randomly selected a ‘target’ child.

### Intervention

PMD is a 10-session intervention. IRC staff developed the program by reviewing existing evidence-based programming and soliciting feedback from content experts on the research team; an earlier iteration of the program was influenced in particular by concepts included in the Nurturing Parenting Program (Bavolek *et al*. 1983). The local IRC office then held focus groups in all five communities with male and female caregivers, as well as activity-based groups with children, to gather information about common parenting practices and roles, including those related to education and health. To inform components of the intervention on harsh parenting, additional conversations with caregivers and community leaders were held to gather information on specific discipline practices. These data informed both the intervention and assessment tools.

A pair of lay Liberian facilitators was trained to lead each weekly 2-hour session with groups of 20–35 caregivers. This group size is larger than that of many other parenting programs but allowed the IRC to serve more people with fewer resources; this is important for scalability in low-resource settings without external funding. This group size also had proven feasible and acceptable in previous piloting of similar interventions by IRC. Sessions were highly interactive with an emphasis on discussion, modeling, and in-session skills practice. The program focused on positive parenting and included briefer components on building cognitive and educational skills and malaria prevention behaviors (see Table 1). Facilitators visited the home of each caregiver once during the program to reinforce skills. The IRC provided caregivers with an incentive of approximately $1.50 USD per session as is customary of programs offered by non-governmental organizations in this setting. Caregivers were permitted to attend the program with a spouse or co-caregiver.

**Table 1.** Summary of the ‘Parents Make the Difference’ curriculum

Session	Topic
1	*Introduction to nurturing and positive parenting*
	Welcome caregivers and provide an overview of the program. Explore the caregivers’ own childhoods and their experience of parenting. Discuss their goals for their children
2	*Childhood development and appropriate expectations*
	Provide psychoeducation regarding child development and age-appropriate expectations. Review the influence of environmental factors on child cognitive, emotional, and social development. Introduce the concept of praise and how it can promote positive child functioning
3	*Communication with children and empathetic listening* Discuss effective communication strategies with young children and the use of play to teach and communicate with children. Introduce the concept of empathy and the importance of mutual respect between parents and children
4	*Discipline with dignity*
	Discuss the importance of positive discipline. Review and practice positive behavior management skills, including praise, ignoring, and time-out
5	*Activities to promote academic readiness*
	Review and practice simple activities, such as story-telling and word games, that can promote a child's cognitive and academic development. Encourage parental involvement in their child's school activities and academics
6	*Malaria prevention*
	Review the causes and dangers of malaria and why children are especially vulnerable. Discuss prevention methods and appropriate response to early symptoms
7	*Academic games: making learning fun!*
	Build on Session 5 and review and practice more specific academic games, emphasizing early math skills and fine motor skills
8	*Establishing routines and house rules*
	Discuss the importance of positive routines and rules for young children
9	*Parent self-care and stress management*
	Review the concept of parent self-care and stress management. Introduce basic relaxation exercises and the concept of positive thinking
10	*Wrap Up: Summarize lessons learned and celebrate successes!*
	Summarize the highlights of the program and praise caregivers for their positive progress

Note. Each session lasted approximately 2 hours. Sessions were designed to be highly interactive with a strong emphasis on discussion, modeling, and in-session practice of skills.

### Research design

**Fig. 1.**
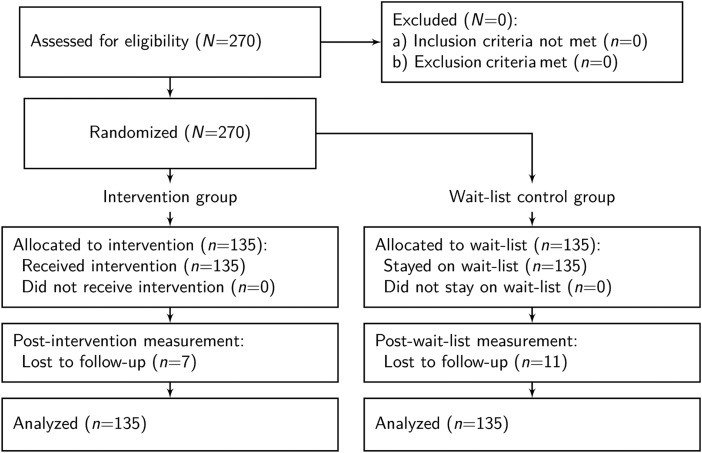
Participant flow diagram.

This was a pragmatic, parallel-group, individually-randomized superiority trial. Eligible primary caregivers were randomized into a treatment or wait-list control group with a 1:1 allocation to treatment ratio, stratified by community. A participant flow diagram is displayed in Fig. 1. With power of 0.80, *α* of 0.05, equal allocation of participants to treatment and control conditions, and an assumption of minimal attrition, this study was designed to detect a standardized effect of at least 0.30.

### Procedures

A team of trained Liberian enumerators conducted a baseline survey of caregivers and target children in September 2013. After the survey, we held a public lottery in each community and randomly assigned eligible caregivers (without replacement) to immediate or delayed treatment. IRC staff delivered the 10-session intervention to caregivers in the immediate treatment group over the course of 13 weeks in early 2013. Approximately 1 month after the final session, we surveyed caregivers in both groups prior to providing the intervention to the waitlist control group. Enumerators administered all surveys at participants’ homes and captured responses with Android smart-phones. During the same home visit, enumerators also asked caregivers and their children to play together for 5 min to complete an observational measure of parent–child interactions. Enumerators were blinded to treatment assignment unless the caregiver divulged this information.

The research protocol was reviewed and approved by local community advisory boards in Liberia from all communities and the Duke University Institutional Review Board. This study was pre-registered at ClinicalTrials.gov (registration number omitted).

### Measures

Prior to data collection, the local research team evaluated the study instruments through systematic piloting and cognitive interviewing. To do this, the local research team administered the items to caregivers and children in the community and asked participants to explain their responses to assess understandability and to advise on clearer ways to ask or translate the items. This led to changes in the core content of the questions, as well as to changes in the translations from American to Liberian English.

### Primary outcomes

#### Parenting behavior

We asked caregivers to self-report on their use of six harsh discipline practices – such as whipping, slapping, and yelling at the child – in the 4 weeks prior to the survey on a scale of 0 (‘never’) to 4 (‘almost every day’). Items were adapted from the Discipline Module of the Multiple Indicator Cluster Survey (UNICEF, 2005). Responses were averaged to create a composite index. We also asked caregivers to rate their use of four positive behavior management strategies: ‘time out’, teaching rules, praising compliant behavior, and asking the child to stop bad behavior. The ‘praise’ item was rated on a different 0–3 scale, so all positive behavior management items were standardized before summing to create a composite that was then standardized. Caregivers also provided an open-ended description of their response the last time the child misbehaved. Enumerators coded responses to match 15 possible behaviors; non-matching responses were coded as ‘other’.

#### Caregiver–child interactions

We asked caregivers and children to rate their experience of four behaviors: spending time together, playing together, talking together, and praise. Caregivers rated their tendency to do these behaviors by indicating their position on a 10-step ladder where the 10th step represents maximum engagement. Children were asked whether the caregiver did these things in the 7 days prior to the survey. To reduce difficulty, enumerators first asked about the behavior using simplified wording and phrasing in the form of a ‘yes/no’ question. If the child responded ‘yes’, the enumerator then asked how much: ‘a small amount’ (1), ‘a medium amount’ (2), or ‘a lot’ (3). ‘No’ responses were scored as 0. We created composite scores for caregivers and children by averaging responses to each set of items.

### Secondary outcomes

#### Communication

Audio recordings from the 5 min caregiver-child play observation were transcribed by Liberians and coded by trained assistants (American) using the Dyadic Parent–Child Interaction Coding System (DPICS-III; Eyberg *et al*. 2005). DPICS allows for the classification of parent verbalizations that serve as markers of parent-child relationship quality. Specific categories include: neutral talk, praise, reflection, behavior description, questions, commands, and negative talk. Assistants were trained according to the DPICS clinical coding manual (Chase & Eyberg, 2005; Fernandez *et al*. 2005) and reached 80% agreement with the trainer before coding independently; 10% of all transcripts were randomly selected to be re-coded by a second rater.

#### Child cognitive abilities

We developed a short battery of cognitive tasks similar to those on standardized developmental and cognitive measures. Domains included receptive language, expressive language, verbal comprehension, verbal fluency, and numeracy. Scores on language domains were standardized and summed to create an overall language ability composite that was then standardized.

#### Child wellbeing

The Strengths and Difficulties Questionnaire (SDQ) is a 25-item behavioral screening questionnaire about children 3–17 years old (Goodman, 1997). We administered three subscales of the SDQ to caregivers: hyperactivity/inattention, emotional symptoms, and conduct problems.

#### Malaria prevention behaviors

We also asked caregivers whether the household owned a mosquito net, whether the target child slept under the net the night before the survey, and other patterns of use.

#### Empirical strategy

We calculated intention-to-treat (ITT) estimates of the average treatment effect, *θ*_ITT_, using the following ordinary least squares specification:
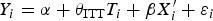
where *Y*_*i*_ is an outcome of interest for person *i* measured 1 month after the treatment group completed the program; *T*_*i*_ is an indicator for assignment to treatment (1 = treatment; 0 = wait-list control); *X*_*i*_ is a vector of community strata dummies and baseline covariates. In the main specification, all missing data were imputed with median values.

In addition to looking at the average treatment effects, we also examined treatment heterogeneity via subgroup analyses (Longford, 1999) and quantile regression (Koenker, 2005). Subgroup (moderator) analyses allow us to ask whether the treatment was more or less effective for some participants. We estimated treatment heterogeneity by interacting assignment to treatment with each baseline covariate we are testing as a potential moderator. Quantile regression allows us to examine the variation in impact across the distribution of outcomes. We did not pre-register heterogeneity analyses, so these results should be viewed as exploratory; however, our selection of potential moderators was guided by similar work (Gardner *et al*. 2010).

To explore the mechanisms through which the intervention might impact more distal outcomes (e.g. child emotional symptoms), we considered two possible post-baseline mediator (or intervening) variables: caregiver-reported use of harsh discipline and caregiver–child positive interactions. We tested a multiple mediator model that includes both potential mediators simultaneously and compute bootstrapped 95% confidence intervals (CI) (adjusted bootstrap percentile method) of the total indirect effect and the component indirect effects of each proposed mediator (Preacher & Hayes, 2008).

We conducted all analyses in R version 3.0.2 (R Core Team, 2013) except for the quantile regressions which we conducted in Stata MP version 12.0 (StataCorp, 2011). Our paper was compiled directly from the raw data and analysis code using RStudio version 0.98.932 (RStudio, 2013) and LATEX MacTeX-2013 distribution (LaTeX3 Project, 2013).

## Results

### Participant characteristics

Children (*n* = 269) and their caregivers (*n* = 270) completed a baseline survey. Figure 1 displays the participant flow diagram. 53% of the child sample was female, and the average child was 5.2 years old. The caregiver sample was also almost evenly divided between males and females (57%), and the average caregiver was 35.5 years old. Table 2 demonstrates that the random assignment to treatment and wait-list control resulted in pre-treatment balance.

**Table 2.** Participant baseline characteristics

Characteristics	Control (*n* = 135)	Treatment (*n* = 135)	*p* value
Caregivers			
Mean age (s.d.)	35.89 (10.80)	35.12 (9.69)	0.538
Female	0.56	0.59	0.624
Married or cohabiting	0.90	0.90	1.000
Christian	0.67	0.69	0.795
Mean household income last 4 weeks (s.d.)^a^	29.88 (47.77)	27.96 (41.11)	0.723
Mean hours worked in typical week (s.d.)	22.54 (20.15)	24.14 (19.12)	0.504
Mean household size (s.d.)	7.13 (3.87)	7.07 (3.15)	0.890
Mean number of dependents under 18 (s.d.)	3.79 (1.99)	3.56 (1.67)	0.290
Biological caregiver of target child	0.84	0.83	0.871
Children			
Mean age (s.d.)	5.16 (1.23)	5.16 (1.06)	1.000
Female (%)	0.54	0.52	0.716
Mean SDQ conduct (s.d.)	4.99 (1.33)	5.17 (1.39)	0.284

SDQ, strengths and difficulties questionnaire

Note. ^a^An exchange rate of 74.2 Liberian Dollars per $1USD (12 September 2012) was used to convert to USD. Self-reported income top-coded at the 99th percentile.

### Treatment compliance

Caregiver attendance was high; at least one caregiver from 98% of households attended all 10 sessions. In some cases, a co-caregiver who did not participate in the surveys attended sessions with, or in place of, the caregiver in the study. These patterns of attendance were not tracked, nor were completed home visits.

### Attrition

The overall attrition rate was 6.7%; 18 children and their caregivers did not participate in the endline survey. As shown in Table A.1 in the Online Appendix, there are no statistically significant baseline differences between found and unfound participants.

### Treatment effects

**Fig. 2.**
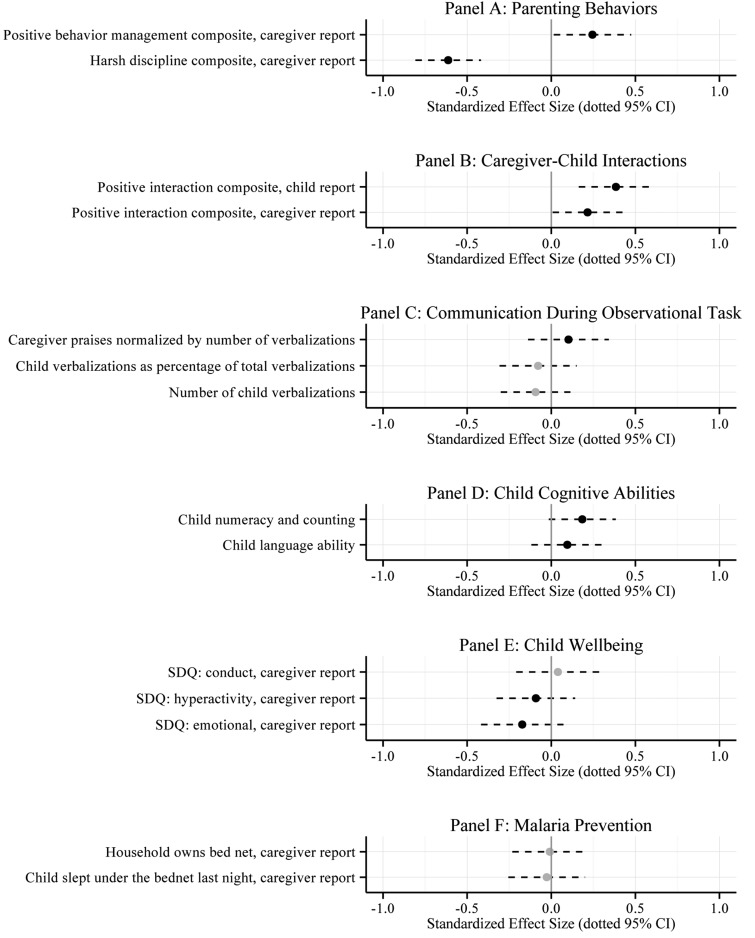
This plot displays standardized results of intention-to-treat (ITT) ordinary least squares (OLS) regressions of each primary and secondary outcome on an indicator of assignment to treatment, stratum fixed effects, and baseline covariates. The point estimates were standardized by dividing the coefficient on assignment by the control group standard deviation (Glass's Δ). Black dots represent point estimates in the hypothesized direction (grey if not in the hypothesized direction). Dotted lines represent 95% confidence intervals (CI).

Program impacts on primary and secondary outcomes are listed in Table 3 and displayed graphically as standardized effect sizes (Glass's Delta) in Fig. 2. Full results are presented in Tables A.2–A.7 in Online Appendix A.

**Table 3.** Average treatment effects: primary and secondary outcomes

		Control			Intent-to-treat (*N* = 270)		
Outcome	Scale (>)-1	Mean -2	s.d.-3	*β*-4	s.e.-5	95% CI-6	Δ-7
Primary outcomes						
Parenting behaviors						
* *Harsh discipline composite, caregiver report	0–4 (−)	0.88	0.80	−0.49	0.08***	(−0.65 to −0.33)	−0.61
* *Positive behavior management composite, caregiver report	*z* (+)	−0.11	1.01	0.25	0.12*	(0.02–0.48)	0.24
Caregiver–child interactions						
* *Positive interaction composite, caregiver report	1–10 (+)	7.69	1.58	0.34	0.17*	(0.01–0.67)	0.22
* *Positive interaction composite, child report	0–3 (+)	1.81	0.82	0.32	0.09***	(0.13–0.5)	0.38
Secondary outcomes						
Communication						
* *Caregiver praises normalized by number of verbalizations	ratio (+)	0.03	0.05	0.01	0.01	(−0.01 to 0.02)	0.10
* *Number of child verbalizations	count (+)	87.57	66.89	−6.24	7.03	(−20.09 to 7.61)	−0.09
* *Child verbalizations as percentage of total verbalizations	0–100 (+)	63.07	24.65	−1.93	2.86	(−7.57 to 3.71)	−0.08
* *Child cognitive abilities						
* * Child language ability	*z* (+)	0.03	0.91	0.09	0.10	(−0.11 to 0.28)	0.10
* * Child numeracy and counting	0–7 (+)	4.85	2.04	0.38	0.21˙	(−0.03 to 0.78)	0.18
Child wellbeing						
* *SDQ: hyperactivity, caregiver report	0–10 (−)	4.21	1.82	−0.17	0.22	(−0.59 to 0.26)	−0.09
* *SDQ: emotional, caregiver report	0–10 (−)	4.16	1.90	−0.33	0.24	(−0.79 to 0.14)	−0.17
* *SDQ: conduct, caregiver report	0–10 (−)	2.09	1.65	0.06	0.21	(−0.34 to 0.47)	0.04
Malaria prevention						
* *Household owns bed net, caregiver report	0–1 (+)	0.90	0.31	0	0.03	(−0.07 to 0.07)	−0.01
* * Child slept under the bed net last night, caregiver report	0–1 (+)	0.87	0.33	−0.01	0.04	(−0.08 to 0.07)	−0.03

SDQ, strengths and difficulties questionnaire

˙*p* < 0.1, *p < 0.05, **p < 0.01, ***p < 0.001.

Note. This table reports average treatment effects (ATE) that are based on a comparison of caregivers assigned to the treatment and control groups. Column 1 lists the scale of each outcome. The character in parentheses indicates the valence of higher values: good (+) or bad (−). Columns 2 and 3 report unadjusted means and standard deviations (s.d.) among the control group. Columns 4 and 5 report the results from an ordinary least squares regression of each outcome on an indicator of assignment to treatment, community fixed effects (omitted), and a vector of baseline covariates (omitted). Column 6 reports the 95% confidence interval (CI) on the estimate reported in column 4. Column 7 reports Glass's Δ, a standardized effect size (ATE/control group s.d.).

### Primary outcomes

#### Parenting behavior

The program led to a 55.5% reduction (−0.49 points on a scale of 0–4) in caregiver-reported use of harsh punishment practices. As shown in Table A.2 (Online Appendix), a greater proportion of caregivers in the treatment group compared with the wait-list control group reported abandoning harsh practices, such as whipping, slapping, beating, and shouting at their children. The standardized treatment effects (Glass's Delta) on harsh parenting practices range from −0.26 to −0.67. We also observe a corresponding significant increase in the use of positive behavior management strategies. This small effect appears to be driven by a significant increase in reported use of time out.

We see the same pattern of results in the open coding of caregivers’ descriptions of how they responded to their child's most recent episode of bad behavior (see Table A.3 in the Online Appendix). On average, a smaller proportion of caregivers in the treatment group described using harsh practices such as yelling and beating, and a larger proportion reported using time out.

#### Caregiver–child interactions

In addition to decreasing caregivers’ use of harsh punishment and increasing the use of positive behavior management strategies, the program also significantly increased positive interactions between caregivers and children. The average caregiver in the treatment group reported a 4.4% increase in positive interactions (0.34 points on a scale of 1–10), while the average child of a caregiver assigned to the treatment group reported a 17.5% increase (0.32 points on a scale of 0–3; standardized effect sizes of 0.22 and 0.38, respectively).

### Secondary outcomes

The average caregiver assigned to the treatment group increased praise and decreased negative talk as a percentage of overall number of caregiver verbalizations during the timed observational play session, but neither effect is statistically significant at conventional levels. Counter to what we hypothesized, we observe a slight decrease in verbalizations among children of caregivers assigned to the treatment group, but this effect is also not statistically significant. We detect a small treatment effect at the 0.10 level on children's receptive vocabulary and numeracy (standardized effect sizes of 0.1 and 0.18, respectively). With the exception of caregiver-reported child conduct problems, child cognitive, and emotional wellbeing indicators move in the hypothesized direction; however, these effects are mostly small and non-significant. The program had no measurable impact on the uptake of malaria prevention behaviors.

### Sensitivity

Bias could potentially result from unobserved baseline imbalance and systematic attrition, so we tested the sensitivity of impacts on primary outcomes. As shown in Table A.8 of the Online Appendix, findings are robust to an alternate specification that excludes baseline covariates. We also created an extreme bound on treatment effects that assumes the ‘worst’ case for missing treatment observations and the ‘best’ case for missing control observations (see table notes for more details). The impact on harsh parenting holds under this extreme assumption, but the impacts on caregiver–child positive interactions and positive behavior management shrink and become non-significant.

### Treatment heterogeneity

We examine treatment heterogeneity in three outcomes – harsh parenting, positive behavior management, and positive interaction – according to four characteristics measured at *baseline*: caregiver gender, child gender, child age, and child conduct problems. Results are presented in Table 4.

**Table 4.** Treatment heterogeneity

Baseline covariates	Outcomes (reporter, scale)					
	Harsh parenting (caregiver, 0–4)		Positive behavior management (caregiver, *z*)		Positive interaction (child, 0–3)	
	*β* (s.e.)		*β* (s.e.)		*β* (s.e.)	
Assigned to treatment	−0.31	(0.12)*	0.49	(0.18)**	0.34	(0.14)*
Female caregiver	0.44	(0.12)***	0.12	(0.18)	0.25	(0.14)˙
Female caregiver × assignment	−0.31	(0.16)˙	−0.43	(0.24)˙	−0.03	(0.19)
Assigned to treatment	−0.60	(0.12)***	0.11	(0.17)	0.33	(0.14)*
Female child	−0.30	(0.12)**	−0.09	(0.17)	0.03	(0.13)
Female child × assignment	0.20	(0.16)	0.26	(0.24)	−0.03	(0.19)
Assigned to treatment	−1.41	(0.37)***	−0.10	(0.55)	−0.22	(0.43)
Child age	−0.08	(0.05)	−0.06	(0.07)	0.01	(0.05)
Child age × assignment	0.18	(0.07)*	0.07	(0.10)	0.10	(0.08)
Assigned to treatment	0.37	(0.42)	0.35	(0.64)	0.22	(0.50)
Child conduct	0.08	(0.03)**	0.00	(0.04)	−0.04	(0.03)
Child conduct × assignment	−0.07	(0.03)*	−0.01	(0.05)	0.01	(0.04)
Observations		270		270		270
Control Mean		0.88		−0.11		1.81

˙*p* < 0.1, *p < 0.05, **p < 0.01, ***p < 0.001.

Note. This table displays the results of a moderation (subgroup) analysis. We examine treatment heterogeneity in three outcomes – harsh parenting, positive behavior management, and positive interaction – according to four characteristics measured at *baseline*: (i) caregiver gender, (ii) child gender, (iii) child age, and (iv) child conduct problems. We estimate treatment heterogeneity by interacting assignment to treatment with each baseline covariate.

With respect to harsh parenting, female caregivers report significantly greater use of harsh punishments, but benefitted twice as much from the intervention compared with male caregivers. Caregivers of boys also reported significantly greater use of harsh punishment, but child gender was not a significant moderator of treatment impact. Child age, however, was a moderator, with caregivers of younger children benefitting more from the intervention. There is also evidence that caregivers of children with more conduct problems at baseline responded more positively. With respect to positive behavior management and children's reports of positive interactions, there was only possible moderation (*p* < 0.10) of caregiver gender on effects on positive behavior management with males showing larger improvements.

Quantile regression results are displayed in Fig. A.1 in the Online Appendix. As shown in Panel B, it appears that the intervention was most effective among caregivers who reported the most frequent use of harsh punishment (−1.31 s.d.)

### Mediation analysis

The intervention's theory of change suggests that teaching caregivers the importance of positive caregiver–child interaction, the ineffectiveness and potential negative impact of harsh discipline strategies, and the utility of positive behavior management approaches – all in the context of modeling and practice – will lead to measureable change on these intermediate caregiver/relationship outcomes, which in turn will improve child outcomes. As reported above, we observe statistically significant impacts on the intermediate outcomes, but small and generally non-significant results for child outcomes such as conduct problems, language ability, and emotional symptoms. While the program impact was non-significant, we can use mediation analysis to explore the mechanism through which the program may impact child outcomes (O'Rourke & MacKinnon, 2015).

As shown in Fig. A.2 in the Online Appendix, child emotional problems appear to be partially mediated by harsh discipline and positive caregiver–child interactions. The total indirect effect is −0.13 s.d. and the 95% CI excludes 0. Both mediators appear to contribute roughly the same amount to the indirect effect. There is also a significant indirect effect of these two mediators on child language. The total indirect effect is 0.13 s.d. We also tested for moderated mediation since we found that caregiver gender is a potential moderator, but we found no evidence for this.

## Discussion

This randomized trial examined the feasibility and impact of a parenting skills intervention for caregivers of young children in Liberia. Findings add to the scarce but growing literature on the impacts of parenting interventions in low-income countries and post-conflict settings. This study focuses on children ages 3–7, an age range well-represented in parenting literature in high-income countries but less so in other parts of the world.

Results show that the PMD reduced caregivers’ use of harsh physical and verbal punishment strategies and increased their use of positive behavior management at 1-month follow-up. The program also improved caregiver–child interactions – according to caregiver and child report – but no significant effects were found on child-level outcomes or malaria prevention behaviors.

The effects of PMD on harsh punishment were the most robust findings, with medium-sized effects on common discipline strategies, including beating, whipping, and shouting. This is comparable with effects of similar programs in the USA. In their review of parenting interventions in low- and middle-income countries, Knerr *et al*. (2013) identified three studies specifically targeting reductions in harsh parenting behaviors. As examples, results were consistent with those of Oveisi *et al*. (2010) who documented moderate effects on harsh discipline following a physician-led parenting intervention in Iran and larger than the small long-term effect on harsh discipline among Turkish mothers after an early enrichment program Kagitcibasi *et al*. (2001). Thus, our results suggest that a parenting intervention delivered by lay facilitators in a post-conflict setting can be as effective for reducing harsh discipline as many similar programs in other contexts.

PMD also had significant small effects on positive behavior management strategies, such as praising positive behavior and using time out. These were smaller than effects of parenting interventions in high-income countries, though studies reporting the largest effects evaluated interventions for children with documented disruptive behavior problems (Thomas & Zimmer-Gembeck, 2007). Thus, the small impact of PMD on positive behavior management could reflect the fact that we studied the impact of a smaller intervention dose on a non-clinical sample. To bolster effects, interventions like PMD may need to include more intense skills modeling and practice, focus on fewer skills, and take into account more of the potential environmental or cultural factors that may point to additional helpful adaptations.

For positive parent–child interactions, such as playing, talking, and time together, PMD led to modest improvements. This is consistent with the study by Kagitcibasi *et al*. (2001) in Turkey documenting small but significant effects of a parenting intervention on mother involvement and parent-child communication. Several studies in low-income countries also have documented benefits of parenting interventions on positive caregiver–infant interactions (Aboud *et al*. 2009; Knerr *et al*. 2013).

Despite having a positive impact on these proximal caregiver-level outcomes, the PMD program did not lead to significant improvements on the secondary outcomes of children's cognitive and communication skills or emotional or behavioral well-being. This is not entirely surprising given the non-clinical sample, as higher levels of distress and emotional dysregulation among oppositional children at baseline predict greater improvements in child outcomes after parent training (Scott & O'Connor, 2012). These studies have led to the ‘differential susceptibility hypothesis’, which posits that children with higher levels of irritability and emotional lability are more impacted by changes in parenting (Bakermans-Kranenburg *et al*. 2008; Scott & O'Connor, 2012). Thus, while the theory underlying parenting interventions is that changes in parenting will positively impact children's development, in non-clinical samples, parenting changes may not have immediate effects; child outcomes may emerge over time – especially the prevention of negative mental health outcomes (Hermanns *et al*. 2013). If child-level effects are to emerge over time, the mechanism of change might be through decreased harsh parenting and increased positive caregiver–child interactions. The results of our mediation analysis suggest that both might mediate child emotional problems. More positive interactions may also mediate the development of children's cognitive abilities, such as language.

Finally, a foundational goal of this study was to determine the feasibility of implementing a parenting skills intervention in a post-conflict, low-resource setting. Attendance and monitoring data supported both the feasibility and acceptability across the communities. This is encouraging because the intervention was implemented in community settings by lay facilitators – a delivery model that is replicable across diverse settings, including those affected by conflict and scarcity of health professionals.

### Limitations

Limitations are related to measurement challenges, length of follow-up, study design, and implementation. First, the broad age range in child age (3–7 years) meant that we could not rely on standardized cognitive measures, and the study was not powered to examine outcomes by age group. This study was also among the first to use an observational measure of parent–child interactions in this context. American research assistants coded the data, and their unfamiliarity with Liberian English vernacular may have contributed to the limited results in this domain. Second, endline measurement 1-month post-intervention may not have allowed sufficient time for potential positive child outcomes to emerge and did not allow for examination of the maintenance of change over time. Third, related to design, it is possible that there was contamination between the groups, which could have attenuated the observed treatment effects. Also, because we used a waitlist control condition, we do not have a time and attention control condition to conclude that effects were due to this specific intervention. Lastly, attendance tracking did not specify whether another family member replaced the enrolled participant at any given session, meaning that attendance rates are at the household level.

### Conclusions and future directions

Results support the feasibility of implementing PMD in a low-income post-conflict setting to reduce harsh treatment of children. PMD was implemented in community-based settings with lay facilitators, a model that could be replicated by aid organizations, governments, or community-level mechanisms.

Future implementations of PMD or similar programs should evaluate modifications that may further increase impact. Live practice with children has emerged as one of the most effective treatment components for children with disruptive behavior disorders (Kaminski *et al*. 2008) and could also enhance outcomes in non-clinical populations. This could be integrated into PMD home visits. However, as home visits are a relatively expensive component, their contribution to the intervention effects and cost-effectiveness would need to be explored.

Given the null results on malaria prevention and cognitive outcomes, it is clear that more work is needed to determine whether and how to integrate parenting, health, and early education interventions. These are key outcomes for young children, and all are caregiver-driven. The challenge is to develop interventions that have enough depth, while also maintaining a reasonable scope and length.

A priority for future study is to evaluate the long-term effects of prevention-focused parenting interventions to determine whether they influence the future development of emotional, behavioral, or cognitive problems throughout childhood and adolescence. Future studies also should examine impacts of PMD with caregivers of children with clinical levels of behavioral or emotional difficulties. These could compare effects of the intervention for clinical *v.* non-clinical samples or compare variants of the program for children and caregivers with varying levels of concerns.

## Supplementary material

For supplementary material accompanying this paper visit http://dx.doi.org/10.1017/gmh.2015.12. click here to view supplementary material
