# Supplementary material for: Parents make the difference: a randomized-controlled trial of a parenting intervention in Liberia
Source: Glob Ment Health (Camb). 2015 Aug 4;2:e15. doi: 10.1017/gmh.2015.12 (PMC5269617; doi:10.1017/gmh.2015.12)
Supplement: Supplementary file 1 [file S2054425115000126sup001.pdf]

Appendix A. Supplementary Tables and Figures

Table A.1: Comparison of baseline characteristics among found and unfound participants at endline

Characteristics	Found	Unfound	p-value
Caregivers			
N	252	18	
Assigned treatment	0.51	0.39	0.331
Mean age (SD)	35.52 (10.25)	35.28 (10.53)	0.923
Female	0.58	0.50	0.534
Married or cohabiting	0.89	0.94	0.490
Christian	0.69	0.56	0.237
Mean household income last 4 weeks (SD)†	29.43 (44.13)	21.80 (50.18)	0.483
Mean hours worked in typical week (SD)	23.75 (19.60)	17.61 (19.61)	0.200
Mean household size (SD)	7.13 (3.55)	6.78 (3.28)	0.685
Mean number of dependents under 18 (SD)	3.64 (1.81)	4.11 (2.25)	0.297
Biological caregiver of target child	0.84	0.78	0.514
Children			
N	251	18	
Assigned treatment	0.50	0.53	0.803
Mean age (SD)	5.17 (1.15)	5.00 (1.06)	0.564
Female (%)	0.52	0.65	0.318
Mean SDQ conduct (SD)	5.07 (1.38)	5.24 (1.09)	0.631

Note. † An exchange rate of 74.2 Liberian Dollars per $1USD (September 12, 2012) was used to convert to USD. Self-reported income top-coded at the 99th percentile.

Table A.2: Average treatment effects: Parenting

		Control		Intent-to-Treat (N=270)			
Outcome	Scale (>) (1)	Mean (2)	SD (3)	β (4)	SE (5)	95%CI (6)	Δ (7)
Harsh discipline composite†	0-4 (-)	0.88	0.80	-0.49	0.08***	(-0.65 to -0.33)	-0.61
Whipped child last 4 weeks	0-1 (-)	0.50	0.50	-0.31	0.05***	(-0.42 to -0.21)	-0.62
Whipped child with hand last 4 weeks	0-1 (-)	0.34	0.48	-0.21	0.05***	(-0.3 to -0.11)	-0.43
Whipped child with object last 4 weeks	0-1 (-)	0.15	0.36	-0.09	0.04*	(-0.16 to -0.02)	-0.26
Slapped child on butt with hand last 4 weeks	0-1 (-)	0.36	0.48	-0.20	0.05***	(-0.3 to -0.1)	-0.42
Beat child last 4 weeks	0-1 (-)	0.53	0.50	-0.34	0.05***	(-0.44 to -0.23)	-0.67
Shouted at child last 4 weeks	0-1 (-)	0.84	0.37	-0.24	0.05***	(-0.35 to -0.14)	-0.65
Positive behavior management composite	z (+)	-0.11	1.01	0.25	0.12*	(0.02 to 0.48)	0.24
Used time out last 4 weeks	z (+)	-0.14	0.95	0.23	0.12*	(0 to 0.46)	0.24
Taught rules about behavior last 4 weeks	z (+)	0.02	0.98	0.07	0.12	(-0.17 to 0.3)	0.07
Asked child to stop behavior in last 4 weeks	z (+)	-0.01	1.00	0.06	0.12	(-0.17 to 0.29)	0.06
Praised child last 4 weeks	z (+)	-0.03	1.02	0.18	0.12	(-0.06 to 0.41)	0.17
Praise is bad for children	1-4 (-)	3.61	0.75	0.07	0.08	(-0.1 to 0.24)	0.10
Sometimes harsh punishment is the only option	1-4 (-)	2.24	1.20	0.01	0.15	(-0.28 to 0.3)	0.01
Ladder of perceived ability to control child behavior	1-10 (+)	8.73	1.54	-0.18	0.21	(-0.6 to 0.24)	-0.12
I am bringing up my child well	1-4 (+)	3.89	0.32	0.09	0.03**	(0.03 to 0.15)	0.29

· $p<0.1$, * $p<0.05$, ** $p<0.01$, *** $p<0.001$

† Items in Harsh Discipline composite are indented below. Items are dichotomized (“ever”==1) in this table, but the full range of responses (0-4) was used to create average composite.

Note. This table reports average treatment effects that are based on a comparison of caregivers assigned to the treatment and control groups. Column 1 lists the scale of each outcome. The character in parentheses indicates the valence of higher values: good (+) or bad (-). Columns 2 and 3 report unadjusted means and standard deviations among the control group. Columns 4 and 5 report the results from an OLS regression of each outcome on an indicator of assignment to treatment, community fixed effects (omitted), and a vector of baseline covariates (omitted). Column 6 reports the 95 percent confidence interval on the estimate reported in Column 4. Column 7 reports Glass's Δ, a standardized effect size (ATE/control group SD).

Table A.3: Average treatment effects: Open coding of parenting strategies

		Control		Intent-to-Treat (N=270)			
Outcome	Scale (>) (1)	Mean (2)	SD (3)	β (4)	SE (5)	95%CI (6)	Δ (7)
Beat body	0-1 (-)	0.41	0.49	-0.32	0.05***	(-0.41 to -0.22)	-0.65
Slapped on the face	0-1 (-)	0.03	0.17	-0.03	0.01*	(-0.06 to 0)	-0.19
Shouted or yelled	0-1 (-)	0.30	0.46	-0.19	0.05***	(-0.28 to -0.09)	-0.41
Denied food	0-1 (-)	0.13	0.34	-0.07	0.04*	(-0.14 to 0)	-0.22
Locked out of the house	0-1 (-)	0.24	0.43	0.12	0.05*	(0.01 to 0.22)	0.27
Pump tire	0-1 (-)	0.07	0.26	-0.04	0.03	(-0.09 to 0.02)	-0.15
Asked to stop behavior	0-1 ( )	0.36	0.48	-0.17	0.05**	(-0.28 to -0.06)	-0.35
Time out	0-1 (+)	0.17	0.38	0.12	0.05*	(0.01 to 0.22)	0.31
Took away privledge/activity	0-1 (+)	0.15	0.36	-0.03	0.04	(-0.11 to 0.06)	-0.08
Advised	0-1 (+)	0.67	0.47	-0.04	0.06	(-0.15 to 0.07)	-0.09
Put to bed	0-1 ( )	0.01	0.12	0.04	0.02·	(0 to 0.09)	0.35
Other	0-1 (-)	0.03	0.17	0.01	0.02	(-0.03 to 0.06)	0.08

· p<0.1, * p<0.05, ** p<0.01, *** p<0.001

Note. This table reports average treatment effects that are based on a comparison of caregivers assigned to the treatment and control groups. Column 1 lists the scale of each outcome. The character in parentheses indicates the valence of higher values: good (+) or bad (-). Columns 2 and 3 report unadjusted means and standard deviations among the control group. Columns 4 and 5 report the results from an OLS regression of each outcome on an indicator of assignment to treatment, community fixed effects (omitted), and a vector of baseline covariates (omitted). Column 6 reports the 95 percent confidence interval on the estimate reported in Column 4. Column 7 reports Glass's Δ, a standardized effect size (ATE/control group SD).

Table A.4: Average treatment effects: Positive interactions

		Control		Intent-to-Treat (N=270)			
Outcome	Scale (>) (1)	Mean (2)	SD (3)	β (4)	SE (5)	95%CI (6)	Δ (7)
Positive interaction composite, caregivers	1-10 (+)	7.69	1.58	0.34	0.17*	(0.01 to 0.67)	0.22
Ladder: time spent with child past week	1-10 (+)	7.57	2.17	0.28	0.24	(-0.19 to 0.75)	0.13
Ladder: played with child past week	1-10 (+)	7.07	2.67	0.79	0.28**	(0.23 to 1.34)	0.29
Ladder: talked with child past week	1-10 (+)	7.81	2.07	0.08	0.23	(-0.37 to 0.53)	0.04
Ladder: praised child past week	1-10 (+)	8.40	1.75	0.20	0.21	(-0.21 to 0.6)	0.11
Positive interaction composite, children	0-3 (+)	1.81	0.82	0.32	0.09***	(0.13 to 0.5)	0.38
Spent time with caregiver past week	0-3 (+)	1.80	1.15	0.27	0.13*	(0.01 to 0.53)	0.23
Played with caregiver at home past week	0-3 (+)	1.87	1.14	0.22	0.14	(-0.04 to 0.49)	0.20
Talked with caregiver past week	0-3 (+)	1.96	1.21	0.39	0.13**	(0.13 to 0.66)	0.33
Praised by caregiver past week	0-3 (+)	1.65	1.18	0.46	0.13***	(0.2 to 0.73)	0.39

· $p<0.1$, * $p<0.05$, ** $p<0.01$, *** $p<0.001$

Note. This table reports average treatment effects that are based on a comparison of caregivers assigned to the treatment and control groups. Column 1 lists the scale of each outcome. The character in parentheses indicates the valence of higher values: good (+) or bad (-). Columns 2 and 3 report unadjusted means and standard deviations among the control group. Columns 4 and 5 report the results from an OLS regression of each outcome on an indicator of assignment to treatment, community fixed effects (omitted), and a vector of baseline covariates (omitted). Column 6 reports the 95 percent confidence interval on the estimate reported in Column 4. Column 7 reports Glass's Δ, a standardized effect size (ATE/control group SD).

Table A.5: Average treatment effects: Communication

		Control		Intent-to-Treat (N=270)			
Outcome	Scale (>) (1)	Mean (2)	SD (3)	β (4)	SE (5)	95%CI (6)	Δ (7)
Ladder: Able to understand childś speech	1-10 (+)	8.57	1.83	0.38	0.20·	(0 to 0.77)	0.21
Ladder: Frequency of childś verbalizations	1-10 (+)	7.40	2.70	0.21	0.33	(-0.43 to 0.85)	0.08
Number of caregiver verbalizations	count (+)	36.51	22.63	-0.09	2.78	(-5.56 to 5.38)	-0.00
Caregiver praises normalized by number of verbalizations	ratio (+)	0.03	0.05	0.01	0.01	(-0.01 to 0.02)	0.10
Caregiver negative talk normalized by number of verbalizations	ratio (-)	0.02	0.03	-0.01	0.00·	(-0.01 to 0)	-0.20
Number of child verbalizations	count (+)	87.57	66.89	-6.24	7.03	(-20.09 to 7.61)	-0.09
Child verbalizations as percentage of total verbalizations	0-100 (+)	63.07	24.65	-1.93	2.86	(-7.57 to 3.71)	-0.08

· $p<0.1$, * $p<0.05$, ** $p<0.01$, *** $p<0.001$

Note. This table reports average treatment effects that are based on a comparison of caregivers assigned to the treatment and control groups. Column 1 lists the scale of each outcome. The character in parentheses indicates the valence of higher values: good (+) or bad (-). Columns 2 and 3 report unadjusted means and standard deviations among the control group. Columns 4 and 5 report the results from an OLS regression of each outcome on an indicator of assignment to treatment, community fixed effects (omitted), and a vector of baseline covariates (omitted). Column 6 reports the 95 percent confidence interval on the estimate reported in Column 4. Column 7 reports Glass's Δ, a standardized effect size (ATE/control group SD).

Table A.6: Average treatment effects: Child abilities and well-being

		Control		Intent-to-Treat (N=270)			
Outcome	Scale (>) (1)	Mean (2)	SD (3)	β (4)	SE (5)	95%CI (6)	Δ (7)
Child language ability	z (+)	0.03	0.91	0.09	0.10	(-0.11 to 0.28)	0.10
Receptive vocabulary	z (+)	-0.03	1.01	0.22	0.11·	(0 to 0.44)	0.21
Expressive vocabulary	z (+)	0.05	0.86	-0.03	0.10	(-0.21 to 0.16)	-0.03
Story comprehension	z (+)	0.10	0.88	-0.05	0.10	(-0.25 to 0.16)	-0.05
Verbal fluency	z (+)	-0.03	0.91	0.02	0.11	(-0.19 to 0.23)	0.02
Child numeracy and counting	0-7 (+)	4.85	2.04	0.38	0.21·	(-0.03 to 0.78)	0.18
SDQ: hyperactivity (care)	0-10 (-)	4.21	1.82	-0.17	0.22	(-0.59 to 0.26)	-0.09
SDQ: emotional (care)	0-10 (-)	4.16	1.90	-0.33	0.24	(-0.79 to 0.14)	-0.17
SDQ: conduct (care)	0-10 (-)	2.09	1.65	0.06	0.21	(-0.34 to 0.47)	0.04

· p<0.1, * p<0.05, ** p<0.01, *** p<0.001

Note. This table reports average treatment effects that are based on a comparison of caregivers assigned to the treatment and control groups. Column 1 lists the scale of each outcome. The character in parentheses indicates the valence of higher values: good (+) or bad (-). Columns 2 and 3 report unadjusted means and standard deviations among the control group. Columns 4 and 5 report the results from an OLS regression of each outcome on an indicator of assignment to treatment, community fixed effects (omitted), and a vector of baseline covariates (omitted). Column 6 reports the 95 percent confidence interval on the estimate reported in Column 4. Column 7 reports Glass's Δ, a standardized effect size (ATE/control group SD).

Table A.7: Average treatment effects: Malaria prevention

		Control		Intent-to-Treat (N=270)			
	Scale (>)	Mean	SD	β	SE	95%CI	Δ
Outcome	(1)	(2)	(3)	(4)	(5)	(6)	(7)
Household owns bed net	0-1 (+)	0.90	0.31	-0.00	0.03	(-0.07 to 0.07)	-0.01
Someone slept under the bednet last night	0-1 (+)	0.84	0.37	-0.01	0.04	(-0.09 to 0.08)	-0.02
Child slept under the bednet last night	0-1 (+)	0.87	0.33	-0.01	0.04	(-0.08 to 0.07)	-0.03
Used bednet when child went to bed	0-1 (+)	0.82	0.38	0.03	0.04	(-0.06 to 0.12)	0.07
Enumerator observed net hanging	0-1 (+)	0.99	0.09	-0.02	0.02	(-0.05 to 0.01)	-0.24

· $p<0.1$, * $p<0.05$, ** $p<0.01$, *** $p<0.001$

Note. This table reports average treatment effects that are based on a comparison of caregivers assigned to the treatment and control groups. Column 1 lists the scale of each outcome. The character in parentheses indicates the valence of higher values: good (+) or bad (-). Columns 2 and 3 report unadjusted means and standard deviations among the control group. Columns 4 and 5 report the results from an OLS regression of each outcome on an indicator of assignment to treatment, community fixed effects (omitted), and a vector of baseline covariates (omitted). Column 6 reports the 95 percent confidence interval on the estimate reported in Column 4. Column 7 reports Glass's Δ, a standardized effect size (ATE/control group SD).

Table A.8: Sensitivity

	Observations			Specifications: Main		No covariates		Missing 90/10	
Outcome	N (1)	Miss (2)	% Miss (3)	β (4)	SE (5)	β (6)	SE (7)	β (8)	SE (9)
Harsh discipline composite, caregiver report	270	18	6.7	-0.49	0.08***	-0.46	0.08***	-0.39	0.08***
Positive behavior management composite, caregiver report	270	18	6.7	0.25	0.12*	0.24	0.12*	0.10	0.12
Positive interaction composite, caregiver report	270	18	6.7	0.34	0.17*	0.34	0.17*	0.10	0.18
Positive interaction composite, child report	270	25	9.3	0.32	0.09***	0.29	0.09**	0.10	0.10
Caregiver praises normalized by number of verbalizations	270	31	11.5	0.01	0.01	0.00	0.01	-0.01	0.01
Number of child verbalizations	270	30	11.1	-6.24	7.03	-4.47	7.18	-24.55	7.63**
Child verbalizations as percentage of total verbalizations	270	30	11.1	-1.93	2.86	-1.51	2.84	-9.97	3.11**
Child language ability	270	56	20.7	0.09	0.10	0.07	0.10	-0.42	0.12***
Child numeracy and counting	270	27	10.0	0.38	0.21·	0.36	0.22	-0.13	0.23
SDQ: hyperactivity, caregiver report	270	18	6.7	-0.17	0.22	-0.16	0.21	0.14	0.23
SDQ: emotional, caregiver report	270	18	6.7	-0.33	0.24	-0.30	0.23	-0.06	0.24
SDQ: conduct, caregiver report	270	18	6.7	0.06	0.21	0.16	0.21	0.38	0.22·
Household owns bed net, caregiver report	270	18	6.7	-0.00	0.03	-0.01	0.04	-0.06	0.04
Child slept under the bednet last night, caregiver report	270	31	11.5	-0.01	0.04	-0.02	0.04	-0.11	0.04*

· p<0.1, * p<0.05, ** p<0.01, *** p<0.001

Note. This table displays the results of a sensitivity analysis of the average treatment effects according to three different specifications. Columns 1 to 3 report on missing data. Columns 4 and 5 report the coefficients and standard errors from ordinary least squares regressions of the outcomes on an indicator of assignment to treatment, community strata, and a vector of baseline covariates. This is the main specification reported in the manuscript; results in these columns are also reported in Table 3. The second specification removes baseline covariates (Columns 6-7). The third specification matches the main specification (Columns 8-9); however, missing observations in the data were imputed according to conservative bounds that assume the worst case for missing treatment observations and the best case for missing control observations. Missing data were imputed at the 10th percentile for outcomes in which higher scores are better (e.g., positive interactions) and at the 90th percentile for outcomes in which lower scores are better (e.g., harsh discipline) for participants assigned to immediate treatment. This pattern was reversed for missing data from participants assigned to delayed treatment (i.e., control).

Quantile Regression The average caregiver assigned to immediate treatment reported a significant reduction in the use of harsh discipline of -0.61 standard deviations. However, as shown in Panel B of Figure A.1, the intervention had differential effects at the tails of the distribution. Specifically, it appears that the intervention was most effective among the caregivers who reported the most use of harsh punishment practices (-1.31 SD) and least effective for caregivers at the opposite end of the distribution (-0.19 SD). Child-reported positive interactions (Panel A) and caregiver-reported use of positive behavior management strategies (Panel C) were more consistent throughout the distribution.

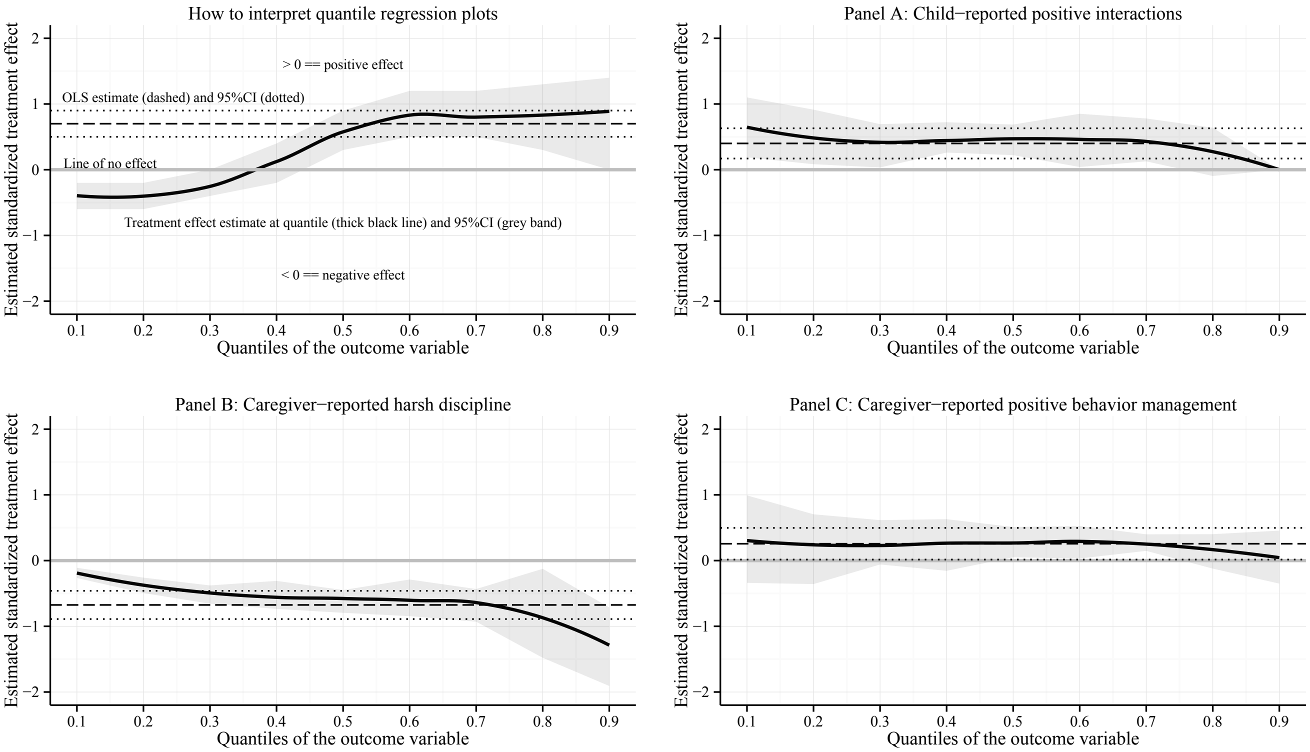

Figure A.1: This figure displays the results of quantile regressions of an outcome on assignment to treatment and a vector of community strata and baseline covariates. Quantiles of the outcome are displayed on the x-axis. The estimated treatment effect is displayed on the y-axis. The solid black line represents the smoothed estimates of the treatment effect at each quantile. This line is surrounded by a 95 percent confidence interval shaded in gray. Also shown in the plot is the average treatment effect from an ordinary least squares regression (dashed line) and its 95 percent confidence interval (dotted lines).

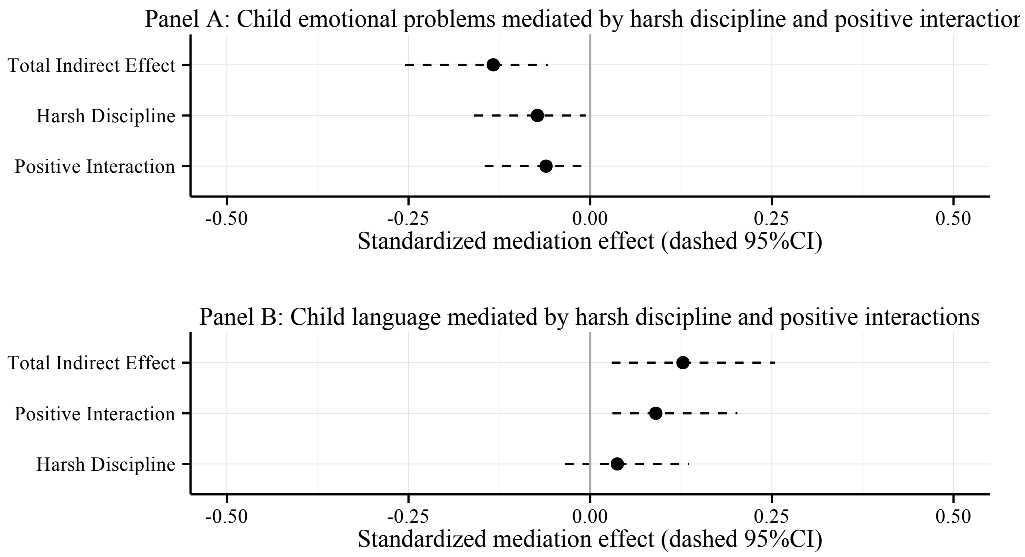

Figure A.2: This figure displays the results of a multiple mediation analysis of two potential mediators, harsh discipline and positive caregiver-child interactions, on two different distal outcomes: child emotional problems and child language. Black dots represent point estimates of the mediation effect. Dotted lines represent bootstrapped 95 percent confidence intervals.
